# Integrating environmental DNA, diving surveys and DNA barcoding to track endangered twaite shad ( *A* *losa fallax*) spawning sites

**DOI:** 10.1111/jfb.70489

**Published:** 2026-05-25

**Authors:** Alessia Ardenghi, Laura Filonzi, Nicolò Bellin, Pietro Maria Rontani, Matthew A. Campbell, Mattia Saccò, Morten E. Allentoft, Daniele Nizzoli, Armando Piccinini, Riccardo Papa, Francesco Nonnis Marzano

**Affiliations:** ^1^ Department of Chemistry, Life Sciences and Environmental Sustainability University of Parma Parma Italy; ^2^ Department Biology University of Puerto Rico of Rio Piedras San Juan Puerto Rico; ^3^ Trace and Environmental DNA (TrEnD) Laboratory. School of Molecular and Life Science Curtin University Bentley Australia; ^4^ Section for GeoGenetics Globe Institute, University of Copenhagen Copenhagen Denmark; ^5^ Gen‐Tech s.r.l. Parma Italy

## Abstract

The twaite shad (*A. fallax*), an anadromous fish species belonging to the Clupeidae family, has experienced population declines throughout Europe. This decline is attributable to a range of anthropogenic pressures, including migration barriers, environmental pollution, habitat degradation, predation by invasive species and unsustainable fishing practices. These factors disrupt the species' lifecycle and ecological balance, leading to significant conservation concerns. Typically, spawning occurs from late spring to early summer, which is when the migration might be up to 400 km upstream from the sea. The demersal eggs hatch relatively quickly, and the larvae start a planktonic life in the river. Traditional monitoring methods have often proved inadequate due to the cryptic nature of *A. fallax*. With the goal of improving the efficacy of current biomonitoring designs, we tested an environmental DNA (eDNA) approach based on 12S rRNA gene Illumina sequencing to monitor *A. fallax* migration and spawning sites in the Po and Taro rivers in Northern Italy. This molecular approach was supplemented by scuba diving observations to visually confirm fish presence and habitat conditions at the same sites. Additionally, the collected larvae were morphologically identified and analysed with DNA barcoding to assess the correct taxonomic assignment of the species. This multi‐level framework confirmed the upstream migration and identified specific spawning sites, as well as the presence of larvae, highlighting the effectiveness of eDNA in detecting *A. fallax* populations. These findings underline the potential of eDNA as a powerful conservation tool for monitoring anadromous fish populations and guiding management strategies.

## 1 INTRODUCTION

In European waters, many diadromous fish species are declining due to impacts associated with anthropogenic activities such as the construction of dams and other migration barriers, along with environmental pollution, habitat loss and overfishing. The twaite shad *Alosa fallax* (Lacépède, 1803), belonging to the family Clupeidae, is an anadromous species with a broad distribution across Europe. The life cycle comprises an adult stage in saltwater habitats with a spawning migration toward rivers and streams. Typically, the spawning occurs in late spring to early summer, between April and June, where the timing depends strictly on the geographic location (Froese & Pauly, 2019). The migration occurs just a few kilometres upstream from river mouths, though spawning migrations as far as 400 km upstream have been observed (Aprahamian et al., 2003). Although some recent population recovery has been observed in specific regions (Magath & Thiel, 2013; Miguel Ángel et al., 2020; Sotelo et al., 2014), historical records indicate declines in several European countries. This sharp reduction in population is the result of its spawning migration behaviour, involving individuals being exposed to threats and several physical obstacles (Limburg & Waldman, 2009). This species was reported in both the Bern Convention (Appendix III) (*Convention on the Conservation of European Wildlife and Natural Habitats*, 1979) and in the Habitats Directive of the European Union (Annexes II and V) (Council of the European Communities) (*Council Directive 92/43/EC on the Conservation of Natural Habitats and of Wild Fauna and Flora*, 1992), and it was considered as vulnerable. Other threats conventionally concerning small populations are the effect of genetic drift and heterozygosity reduction that might expose the species to the loss of genetic variability and limitation of the adaptive evolutionary potential (Frankham, 2005). Conservation planning must also consider the co‐occurrence of demographic declines, hybridization with other species and overlapping geographic niches. When *Alosa fallax* and *A. alosa* spawn in close proximity, they tend to produce reproductively viable hybrids (Jolly et al., 2012). Rougemont et al. (Rougemont et al., 2022) assessed the genetic structure of both *A. fallax* and *A. alosa* across several European localities with microsatellites, inferring a recent re‐contact between species with hybridization and bidirectional gene flow due to shared and constrained spawning sites.

The cryptic behaviour of *Alosa fallax* could make the assessment of its conservation status difficult because it relies strictly on monitoring in certain temporal windows and precise spatial occurrences. Moreover, these spatio‐temporal constraints ideally need to be linked with the identification of local and global threats for through assessment of the conservation status. Environmental DNA (eDNA) represents a powerful monitoring tool that can be used in combination with traditional monitoring techniques to assess the presence or absence of species in a wide variety of ecosystems (Díaz‐Ferguson & Moyer, 2014; Takahashi et al., 2023). eDNA consists of genetic material shed from tissues, excretions and secretions that accumulate in mediums like water, air, soil or sediment (Thomsen & Willerslev, 2015). In the freshwater column, dispersed DNA persists in function of different environmental parameters (Boivin‐Delisle et al., 2021) and might be adsorbed in both organic and inorganic particles. The eDNA detection in temperate water was assessed in a time magnitude less than a month (Dejean et al., 2011). In freshwater ecosystems, eDNA has been successfully applied to detect endangered species (Takahashi et al., 2023), assess metacommunity composition (Pont et al., 2018) and investigate water quality and fish diversity across anthropogenic landscapes (Zhang et al., 2024). eDNA was also used to track seasonal migration trends in the Venice lagoon using High Throughput Illumina sequencing and teleost‐specific 12S rRNA primers (Cananzi et al., 2022). Additionally, it was employed to assess the spatial distribution of *Alosa* spp. following the partial removal of a river impoundment, targeting the cytochrome c oxidase subunit I gene with qPCR (Antognazza et al., 2019).

Our study employed a multi‐level framework to capture the arrival of *A. fallax* from the Adriatic Sea to two spawning sites in the Po River basin (up to 400 km upstream). The results from eDNA analysis and Illumina sequencing of the 12S rRNA gene were compared with citizen science data from local fishers to evaluate the detection power of the method. For result confirmation, an outgroup distant site where the species is absent was taken into consideration. Moreover, after spawning activity, we track the emergence of larvae with scuba diving observations in combination with morphological and DNA barcoding identification. In the present study, the integration between different approaches was intentionally based on the Cytochrome b (*Cytb*) marker rather than on *12S*. *Cytb* was selected because it has been previously validated by our research group as a robust and informative marker for twaite shad (*Alosa fallax*) populations in Italian river systems, allowing direct comparison with existing reference data. This multi‐level approach, which integrated eDNA, morphological identification, and DNA barcoding, successfully revealed the spawning activity of this cryptic anadromous species.

## 2 MATERIALS AND METHODS

### 2.1 Study area

**FIGURE 1:**
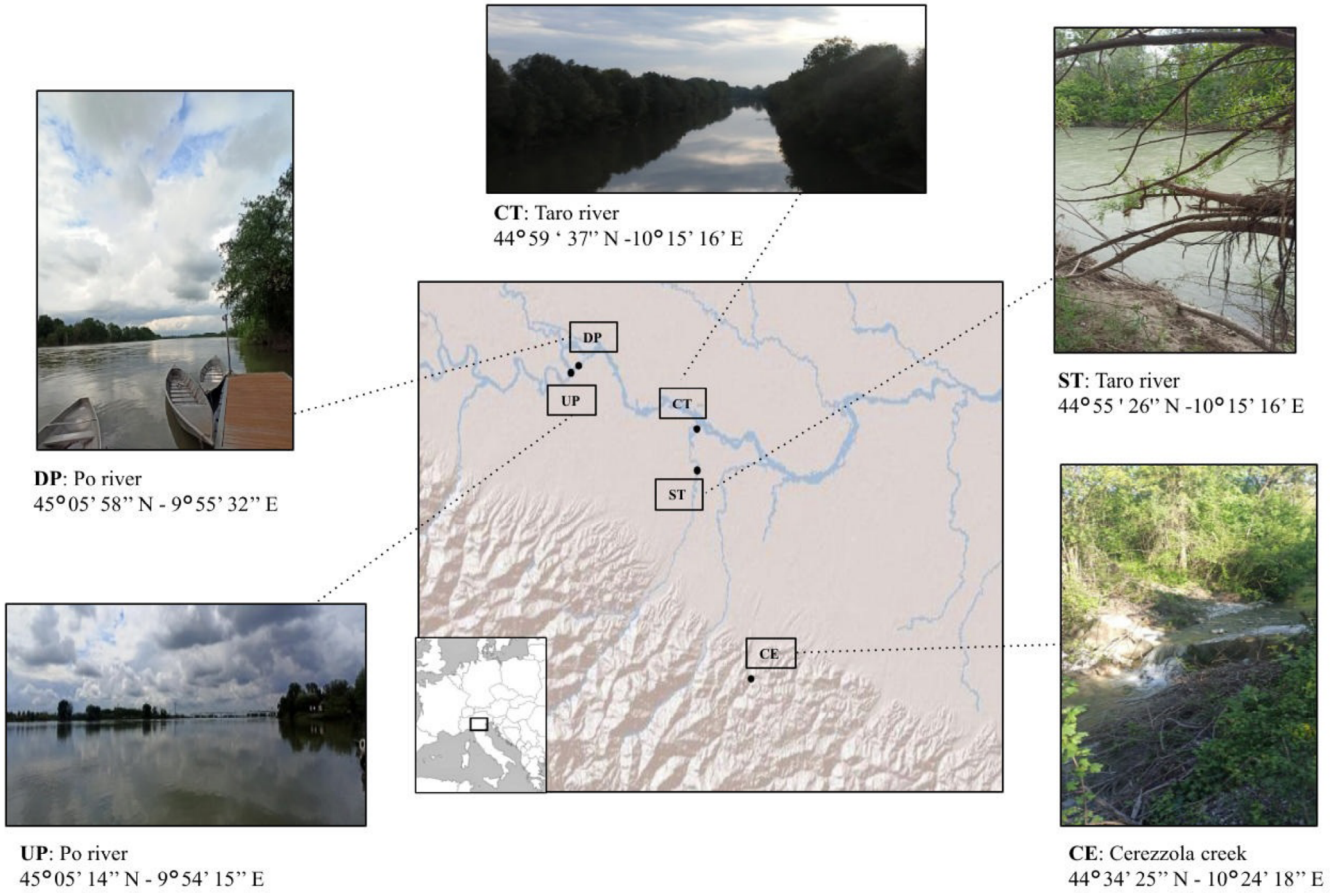
Map of sampling sites for eDNA analysis. The figure illustrates the geographical distribution of the sampling sites used for eDNA analysis to monitor the presence of *Alosa fallax* in the Taro (Sites CT, ST) and Po rivers (UP, DP) and Cerezzola creek (CE).

In this study, five different localities across three rivers of the Emilia Romagna region (Northern Italy) were examined. The first two sites were located in the Taro River, a river that flows through a diverse landscape before joining the Po River. The first site was located at the confluence of the Taro River in the Po River (Site CT, 44**°** 59′ 37″ N to 10**°** 15′ 16″ E, Figure 1 and Table 1) characterized by an extensive floodplain with sediment deposits of fine silt and clay with slower water flow. The second site (Site ST, 44° 55′26″ N to 10**°** 15′ 16′ E, Figure 1 and Table 1) was located 15 km upstream from the river mouth and consists of fast‐flowing, clear waters with a rocky and gravel substrate. Two additional sites were in the Po River near the Isola Serafini hydroelectric dam (Isola Serafini, province of Piacenza, Emilia Romagna, Italy) (Site UP, 45**°** 05′ 14″ N to 9**°** 54′ 15″ E, Figure 1 and Table 1 and Site DP, 45**°** 05′ 58″ N to 9**°** 55′ 32″ E, Figure 1 and Table 1), located respectively upstream and 5 km downstream of the structure, which represents an anthropogenic barrier. A fish passage system has been constructed at this site to facilitate the upstream migration of fish (LIFE11 NAT/IT/000188). A fifth additional site at Cerezzola creek (Enza basin, Site CE, 44**°** 34′ 25″ N to 10**°** 24′ 18″ E, Figure 1 and Table 1) where *A. fallax* occurrence was never detected has been sampled and considered as negative control.

**TABLE 1:** Sampling sites with geographical coordinates. This table provides the IDs, rivers, specific locations and geographical coordinates (latitude and longitude) for the sampling sites involved in the study.

River	Site	Latitude	Longitude	Site code	Date	River condition	Temperature (°C)	pH	*O*_2_ (mg/l)	Specific conductivity (μS/cm at 25°C)
Taro	San Secondo (PR)	44° 55′26″ N	10° 15′ 16′ E	ST1	4/22/2024	High	11.8	8.45	10.7	370
				ST2	5/8/2024	Flood	15.1	8.38	10.41	342
	Taro river mouth (PR)	44° 59′ 37″ N	10° 15′ 16″ E	CT1	4/22/2024	High	13.9	8.42	9.16	422
				CT2	5/8/2024	Flood	17.7	8.38	9.468	380
Po	Upstream Isola Serafini dam (PC)	45° 05′ 14” N	9° 54′ 15″ E	UP1	4/22/2024	High	12.3	8.14	9.88	320
				UP2	5/8/2024	Flood	15.8	8.06	9.47	305
	Downstream Isola Serafini dam (PC)	45° 05′ 58″ N	9° 55′ 32″ E	DP1	4/22/2024	High	12.3	8.13	9.81	323
				DP2	5/8/2024	Flood	15.8	8.12	9.34	305
Cerezzola creek	Cerezzola (PR)	44° 34′ 25″ N	10° 24′ 18″ E	CE1	4/28/2024	‐	‐	‐	‐	‐
				CE2	5/6/2024	‐				

### 2.2 Environmental DNA analysis

#### 2.2.1 eDNA collection, library construction and sequencing

The sampling of the five sites (CT, ST, UP, DP and CE) was repeated in duplicate 1week apart (22nd of April and 8th of May 2024) with the aim of increasing representativeness of the sampling effort and reducing possible dropout events, such as the non‐detection of rare or low‐abundance species. The sampling codes for the first round of sampling are CT_1, ST_1, UP_1, DP_1 and CE_1. For the second round of sampling, the codes are CT_2, ST_2, UP_2, DP_2 and CE_2. The environmental DNA (eDNA) sampling in the Po and Taro rivers sites was conducted during high water levels and flooding conditions caused by heavy rainfall. Samples from the water column were collected through 1‐L high‐density polyethylene (HDPE) sterilized bottles. Water was then filtered through 50‐mL luer‐lock hypodermic Chirana syringes which were fitted with a two‐layer pre‐filter (28‐mm‐diameter nylon sheet and a 28‐mm‐diameter cross‐stitch sheet) to minimize early clogging due to excessive turbidity. Water exiting the syringe was further filtered through 1.2 μM CA enclosed capsule syringe filter (Integra) and filters were preserved in 400 μL of DNA Shield and capped with generic luer‐lock caps. Samples were kept in darkness and at room temperature until further processing. Six replicates per each sampling point (*n* = 5) were collected, 60 samples (30 samples × 2 times). Depending on the turbidity of the water and the conditions of the river, the volume filtered per replicate varied between 100 and 250 mL.

DNA extractions were performed in the Laboratory of Animal Genomics (Department of Chemistry, Life Sciences and Environmental Sustainability, University of Parma) using the Qiagen DNeasy Blood and Tissue Extraction kit (Qiagen, Hilden, Germany) following the manufacturer's protocol. To prevent contamination, all equipment and bench spaces were cleaned before use with 10% sodium hypochlorite solution followed by 70% ethanol and irradiated with UV light for 20 min. DNA quantity was evaluated using the Qubit dsDNA HS (High Sensitivity) Assay Kit (Invitrogen, Waltham, MA, USA) with a Qubit 3.0 Fluorometer (Life Technologies, Waltham, MA, USA). All extractions were stored at −20°C until further processing.

Massively parallel paired‐end sequencing was performed on an Illumina MiSeq platform (2 × 250 bp, San Diego, CA, USA). Amplicons were prepared following the Illumina protocol, which requires fragments to include primer‐binding regions, dual‐index barcodes and adapter sequences for flow cell attachment. The primers 12S tele02 are targeted to amplify a short fragment of about 130–209 bp of the 12S rRNA mtDNA gene of bony fishes (Cananzi et al., 2022); tele02‐F 5′‐AAACTCGTGCCAGCCACC‐3′ and tele02‐R 5′‐GGGTATCTAATCCCAGTTTG‐3′. Illumina overhang adapters were added to these locus‐specific primers to enable sequencing. The first Amplicon PCR was carried out with 27 cycles of a 25‐μL reaction volume containing 12.5 μL 2 × KAPA HiFi HotStart ReadyMix (including DNA polymerase, reaction buffer, dNTPs and MgCl_2_ [at a final concentration of 2.5 mM]) (KAPA Biosystems, Wilmington, MA, USA), 5 μL of each primer (1 μM) and 2.5‐μL template (average DNA concentration of 0.4 ng/μL). The thermal cycle profile after an initial step of denaturation (3 min at 95°C) was as follows: 25 cycles of denaturation at 95°C for 30 s; annealing at 57°C for 30 s; and extension at 72°C for 30 s with a final extension at 72°C for 5 min. The PCR clean‐up has been performed using AMPure XP beads (Beckman Coulter). The second Index PCR was carried out with a 50‐μL reaction volume containing 25 μL 2 × KAPA HiFi HotStart ReadyMix, 10 μL of the unique dual (UD) indexes primer (DNA/RNA UD indexes Set B primer, Illumina), 10‐μL sterile distilled H_2_O and 5.0‐μL template. The thermal cycle profile after an initial 3‐min denaturation at 95°C was as follows: eight cycles of denaturation at 98°C for 30 s, annealing at 55°C for 30 s and extension at 72°C for 30 s with the final extension at the same temperature for 5 min. After purification, the pooled library was normalized to 4 nM, denatured with 0.1 N NaOH and diluted with HT1 buffer (Illumina MiSeq V2 reagent kit) to a final concentration of 8 pM for sequencing. A 30% PhiX spike‐in was included to increase base diversity and improve sequencing quality.

#### 2.2.2 Data processing

Raw FASTQ reads were demultiplexed on the MiSeq platform using Illumina's bcl2fastq software (v2.20). Adapter sequence trimming and read quality control were performed using AdapterRemoval v2 (Schubert et al., 2016). Following this, primer sequences were removed from the cleaned reads using Cutadapt (Martin, 2011), ensuring the elimination of potential primer‐derived biases. The resulting reads were subsequently merged using the USEARCH tool (Edgar, 2010). Zero‐radius operational taxonomic unit (ZOTU) formation and taxonomic assignment were conducted utilizing the eDNAFlow bioinformatics pipeline (Mousavi‐Derazmahalleh et al., 2021), executed on the Setonix HPE Cray EX supercomputer housed at the Pawsey Supercomputing Centre in Kensington, Western Australia. ZOTUs with a minimum abundance threshold of 4 were generated from the merged sequences using the USEARCH unoise3 algorithm to filter out low‐abundance sequences likely to contain errors (Edgar, 2016). These ZOTUs were then queried against the GenBank nucleotide reference database using BLASTN (Altschul et al., 1990), with parameters set at 100% query coverage, 95% identity, an e‐value threshold of 1e‐3 and a maximum of 10 target sequences per query. To further refine the dataset, erroneous ZOTUs were identified and removed using the LULU post‐clustering curation method (linkage of unique lineages units), applying a minimum sequence similarity threshold of 97% (Frøslev et al., 2017). Finally, a custom Python script was employed to assign ZOTUs to their nearest common ancestor (LCA) (Mousavi‐Derazmahalleh et al., 2021). Taxonomic assignments were collapsed to the LCA if the percent identity between two hits with 100% query cover and 95% identity differed by less than 1%. To remove tag jumps, the R package METABAR was employed with the ‘tagjumpslayer’ function, applying a threshold of 0.03 (Zinger et al., 2021). The data were subsequently transformed into a categorical matrix representing four occurrence levels (absence, rare, common and abundant) through a 2‐bit encoding scheme. This discretization process was implemented using a custom R script to facilitate standardized downstream analyses. Subsequently, the categorized data were visualized and clustered by sites and species using the PHEATMAP package, which facilitated the identification of underlying patterns and relationships within the dataset (Kolde, 2019).

#### 2.2.3 Modelling the occurrence of *A. fallax* with environmental variables

The environmental variables specific conductivity (μS/cm at 25°C), pH, temperature (°C) and dissolved oxygen (O_2_ mg/l) were measured using the multiparametric probe AP‐2000 Advanced portable multi‐parameter Aquaprobe in all sites, but CE (the outgroup). The occurrences (presence or absence) of *A. fallax* were modelled within a Bayesian framework considering all the environmental variables (Table 1). An additive logistic model of a Bernoulli family was fitted using 2 chains, 4000 iterations and a warmup (or burn‐in) phase of 2000. After model fitting, the absolute effect sizes of the estimated coefficients were assessed based on the range of their 95% credible intervals. Credibility intervals range that overlaps with 0 indicates uncertainty about the direction of the effect, suggesting that the effect might be null. The Bayesian model was fitted with the R package brms (Bürkner, 2019). Moreover, the most important variables in the model were identified using the p‐direction with the R packages bayesTestR (Makowski et al., 2019). Values of p‐directions above 95% were considered as an indicator of importance of the independent variables in the model and conditional effects were computed with the R packages brms (Bürkner, 2019).

### 2.3 Scuba diving and larvae observation

In the month of June, following eDNA sampling results, scuba diving operations were carried out to highlight the presence of twaite shad larvae at the reproductive sites (ST sites). The scuba diving operator used a closed‐circuit oxygen rebreather, model Castoro C96 Pro (from OMG Italia), which allows for long diving times without bubble emission, and a Nikon D750 camera inside a Subal underwater housing with an Ikelite DS160 flash. Each underwater inspection lasted approximately 30–40 min, depending on water visibility and current conditions.

The larvae were observed and captured at a depth of 1.5–2 m on a pebbly substrate by using a 40 × 40 cm landing net with a fine mesh (1 × 1 mm) on an 80‐cm‐long pole. The strong schooling behaviour of twaite shad larvae greatly facilitated their capture. The operator focused on gravel and pebble substrates, which are known to provide optimal conditions for *A. fallax* spawning and larval development, characterized by higher oxygenation and interstitial stability. Areas dominated by sand or silt were also inspected for comparison. Water visibility during the underwater inspection was approximately 5 m, even at a depth of 15 m. Specimens were collected and transported to the Animal Genomics Laboratory, Department of Chemistry, Life Sciences and Environmental Sustainability, University of Parma (Italy).They were sedated with phenoxyethanol at a concentration of 1 mL per 2 L of water to reduce stress due to the operations necessary for morphological analyses and caudal fin sampling. The larvae were observed using a ZEISS Stemi V8 stereomicroscope with an image detector Canon PowerShot G5. Subsequently, from each individual, a portion (0.200 to 0.300 mm^2^) of the caudal fin was collected from live larvae. Shad larvae were released alive at the same collection site (ST) after tissue sampling and measurements. Fin tissues were preserved in absolute ethanol and then stored at −20°C in the Animal Genomics Laboratory.

All procedures were performed according to the Regional (L. R. 07 novembre 2012, n. 11) and National laws (D. lgs. 27 gennaio 1992, n. 116) preserving animal welfare and health. No relevant ethical request is necessary according to the above cited laws and the applied experimental procedures. In particular, all larvae fish submitted to narcosis were released alive at the end of the procedures.

### 2.4 DNA barcoding for larvae species identification

High molecular weight genomic DNAs were extracted and purified from caudal fin clips of 15 shad larvae using the Wizard genomic DNA Purification kit (Promega, Madison, WI, USA). DNA quality was visually inspected by 1% agarose gel electrophoresis in TAE buffer and by spectrophotometry at 260–280 nm. The extraction procedure yielded not less than 40 ng/μL of HMW (high molecular weight) DNA. Cytochrome b (*Cytb*) gene fragment was amplified using primers Alocytbf1 and Alocytbr1 (Faria et al., 2006), which amplify 515‐bp fragments. Detailed amplification protocol is reported in (Chiesa et al., 2014). Amplicons were separated by 2.5% agarose gel electrophoresis and purified using the Qiagen MinElute PCR Purification Kit (QIAGEN, Venlo, The Netherlands). The quality of the purified sample (1 μl) was visualized in 1.5% agarose gel that yielded clear bands. DNA quantity was evaluated using the Qubit dsDNA HS (High Sensitivity) Assay Kit (Invitrogen, Waltham, MA, USA) with a Qubit 3.0 Fluorometer (Life Technologies, Waltham, MA, USA). Forward and Reverse fragments sequencing was performed by Macrogen Europe Lab (Amsterdam, the Netherlands). The obtained sequences were manually corrected using MEGA7 (Kumar et al., 2018) and compared with those available in the NCBI genomic databases (National Institutes of Health, Bethesda, MD, USA) using the BLAST service (National Institutes of Health, Bethesda, MD, USA). The species level was assigned when the identity rate was greater than 98% (Pardo et al., 2018).

## 3 RESULTS

### 3.1 eDNA analysis

The MiSeq paired‐end sequencing (2 × 250 bp) generated a total of 17.48 million reads, with an average of 87.4% of base calls achieving Phred quality scores of ≥30.0 (Q30; error rate = 0.1%, base call accuracy = 99.9%) (Figures S1 and S2). After demultiplexing and subsequent pre‐processing of the raw data, the sequences were subjected to BLAST searches for taxonomic assignment.

**FIGURE 2:**
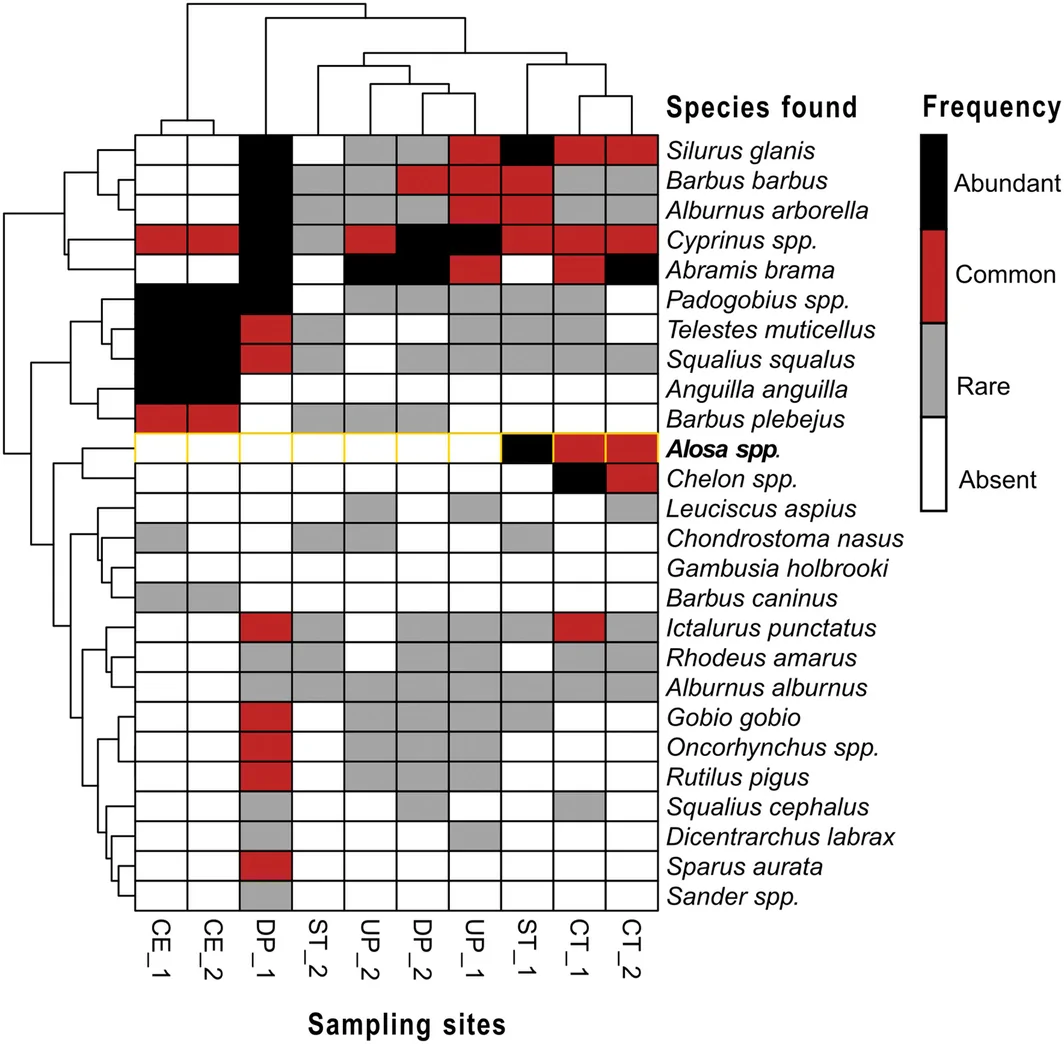
Heat map displaying the distribution of species across the sampling sites ST, CT, UP, DP and CE sampled twice. Colour coding is as follows: white‐species that are absent from the site, grey‐rare species detected at the site, brown‐species that are present at the site and black‐common species frequently found at the site.

A total of 240,029 reads were assigned for analysis. Of these, 191,807 reads (approximately 79.9%) were excluded due to their classification within the families Aves and Mammalia. The remaining 48,222 reads (20.1%) were successfully identified as fish species, with a sequence identity of ≥97%. Read abundance and species richness varied markedly among sites: CE_1 and CE_2 yielded the highest sequencing output (~19,700 reads) but relatively low richness (8–9 species). Intermediate values were observed at ST_1 (4071 reads; 12 species) and UP_2 (912 reads; 13 species), while ST_2 showed both low read counts (395) and reduced richness (10 species). In contrast, DP_1, UP_1 and DP_2 exhibited lower sequencing depth overall (1400–5400 reads) yet the greatest species richness (15–18 taxa). Overall, eDNA analysis identified a varying number of species at each site, with a total of 26 fish species detected across the five sites, each sampled twice (Table 2). *Alosa fallax* was detected at Sites ST and CT (Figure 2). Species were considered present when ≥10 reads were detected across at least two replicates, and taxonomic assignments were retained only when the LCA confidence score was ≥0.9. To facilitate interpretation of abundance patterns, the raw read matrix was transformed into a semi‐quantitative categorical scale, with 0 indicating absence (0 reads), 1 low abundance (1–100 reads), 2 moderate abundance (101–300 reads) and 3 high abundance (>300 reads). A heat map visualizing the relative abundance and presence of different species across all sampled locations highlights the distribution patterns. Notably, *A. fallax* was absent during the second sampling at Site ST (designated ST_2). Furthermore, *A. fallax* was not detected at the UP and DP sites in the Po River, nor at Site CE, where its absence aligns with known distribution patterns. The heat map clustering reveals a distinct separation of the Cerezzola site (CE) from the other sampled locations, indicating a significantly different species composition. This consistent clustering of Cerezzola as an outgroup, despite being sampled twice, underscores its unique ecological characteristics. Conversely, the clustering of the Po River sites (UP, DP) and the Taro River sites (ST, CT) suggests a higher similarity in species composition within each river system. This pattern may reflect similar environmental conditions or hydrological connectivity within each river. The environmental variable with the highest absolute effect size was conductivity (Estimate = 2.36, C.I. _l‐95%_ = 0.39 and C.I._h‐95%_ = 5.63) followed by oxygen (Estimate = −0.18, C.I. _l‐95%_ = −4.88 and C.I._h‐95%_ = 4.51), temperature (Estimate = −0.16, C.I. _l‐95%_ = −4.61 and C.I._h‐95%_ = 4.45) and pH (Estimate = −0.02, C.I. _l‐95%_ = −4.98 and C.I._h‐95%_ = 4.93).

**TABLE 2:** Species detected for site. The table presents the species detected at each sampling site using eDNA analysis. The ‘site identifiers’ column lists the site identifiers, the ‘species counting’ column indicates the number of different species detected at each site and the ‘species found’ column provides the names of the species found at each respective site. The sampling codes for the first round of sampling are CT_1, ST_1, UP_1, DP_1 and CE_1. For the second round of sampling, the codes are CT_2, ST_2, UP_2, DP_2 and CE_2.

Site identifiers	Species counting	Species found
CE_1	9	*Padogobius* spp., *Cyprinus* spp., *Telestes muticellus*, *Squalius squalus*, *Anguilla anguilla*, *Chondrostoma nasus*, *Barbus plebejus*, *Anguilla* spp., *Barbus caninus*.
CE_2	8	*Padogobius* spp., *Cyprinus* spp., *Telestes muticellus, Squalius squalus, Anguilla anguilla, Barbus plebejus, Anguilla* spp., *Barbus caninus*.
CT_1	14	*Padogobius* spp., *Cyprinus* spp., *Telestes muticellus*, *Abramis brama*, *Squalius squalus*, *Alosa fallax*, *Rhodeus amarus*, *Silurus glanis*, *Barbus barbus*, *Alburnus arborella*, *Chelon* spp.,*Ictalurus punctatus*, *Alburnus alburnus*, *Squalius cephalus*
CT_2	12	*Cyprinus* spp., *Abramis brama*, *Squalius squalus*, *Alosa fallax*, *Rhodeus amarus*, *Silurus glanis*, *Barbus barbus*, *Alburnus arborella*, *Chelon* spp., *Ictalurus punctatus*, *Alburnus alburnus*, *Leuciscus aspius*
DP_1	18	*Padogobius* spp., *Cyprinus* spp., *Telestes muticellus*, *Abramis brama*, *Squalius squalus*, *Rhodeus amarus*, *Silurus glanis*, *Barbus barbus*, *Alburnus arborella*, *Ictalurus punctatus*, *Alburnus alburnus*, *Oncorhynchus* spp., *Gobio gobio*, *Rutilus pigus*, *Sparus aurata*, *Sander* spp., *Squalius cephalus*, *Dicentrarchus labrax*
DP_2	15	*Padogobius* spp., *Cyprinus* spp., *Abramis brama*, *Squalius squalus*, *Rhodeus amarus*, *Silurus glanis*, *Barbus barbus*, *Alburnus arborella*, *Ictalurus punctatus*, *Alburnus alburnus*, *Oncorhynchus* spp., *Gobio gobio*, *Rutilus pigus*, *Barbus plebejus*, *Squalius cephalus*
ST_1	12	*Padogobius* spp., *Cyprinus* spp., *Telestes muticellus*, *Squalius squalus*, *Alosa fallax*, *Silurus glanis*, *Barbus barbus*, *Alburnus arborella*, *Ictalurus punctatus*, *Alburnus alburnus*, *Gobio gobio*, *Chondrostoma nasus*
ST_2	10	*Cyprinus* spp., *Telestes muticellus*, *Squalius squalus*, *Rhodeus amarus*, *Barbus barbus*, *Alburnus arborella*, *Ictalurus punctatus*, *Alburnus alburnus*, *Chondrostoma nasus*, *Barbus plebejus*
UP_1	16	*Padogobius* spp., *Cyprinus* spp., *Telestes muticellus*, *Abramis brama*, *Squalius squalus*, *Rhodeus amarus*, *Silurus glanis*, *Barbus barbus*, *Alburnus arborella*, *Ictalurus punctatus*, *Alburnus alburnus*, *Oncorhynchus* spp., *Gobio gobio*, *Rutilus pigus*, *Leuciscus aspius*, *Dicentrarchus labrax*
UP_2	13	*Padogobius* spp., *Cyprinus* spp., *Abramis brama*, *Silurus glanis*, *Barbus barbus*, *Alburnus arborella*, *Alburnus alburnus*, *Oncorhynchus* spp., *Gobio gobio*, *Rutilus pigus*, *Chondrostoma nasus*, *Barbus plebejus*, *Leuciscus aspius*

**FIGURE 3:**
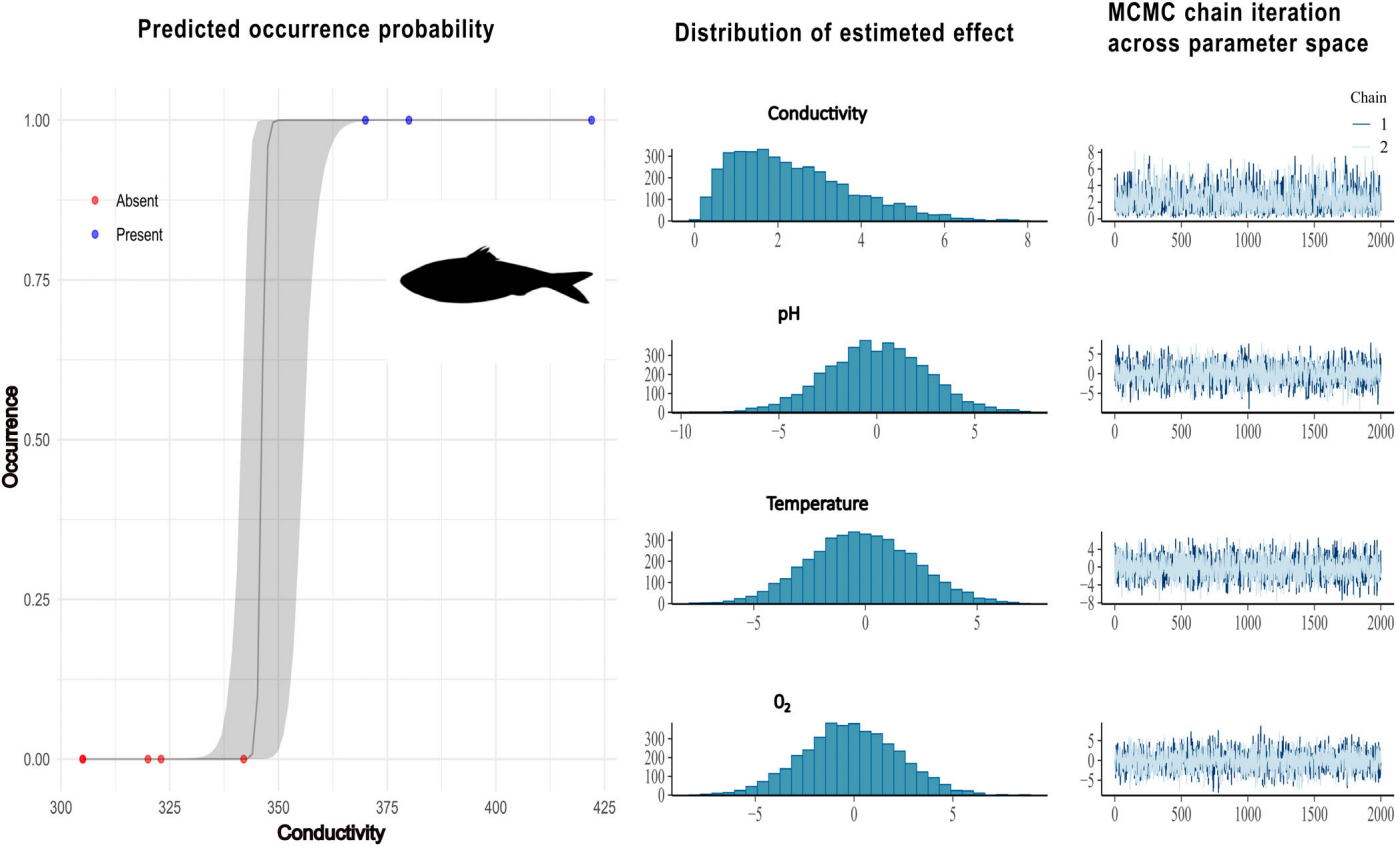
Predicted probability of *A. fallax* occurrence as a function of conductivity (μS/cm) (left). Blue and red points represent presence and absence records, respectively, with the shaded area indicating the 95% credible interval. On the right, posterior distributions of estimated effect sizes (centre) and MCMC trace plots (right) are shown for conductivity, pH, temperature and dissolved oxygen. For each chain, the exploration of the parameters space is shown.

The most important environmental variable identified in the Bayesian model that shaped the occurrence of *A. fallax* was the conductivity (conductivity_p‐direction_ = 100%, Figure 3) with the probability of occurrence that increases for values around 350 μS/cm (Figure 3). The other environmental variables showed no relevant effects (pH_p‐direction_ = 50.4%, temperature_p‐direction_ = 53.2%, 02_p‐direction_ = 52.9%).

### 3.2 Larvae morphological identification and DNA barcoding

**FIGURE 4:**
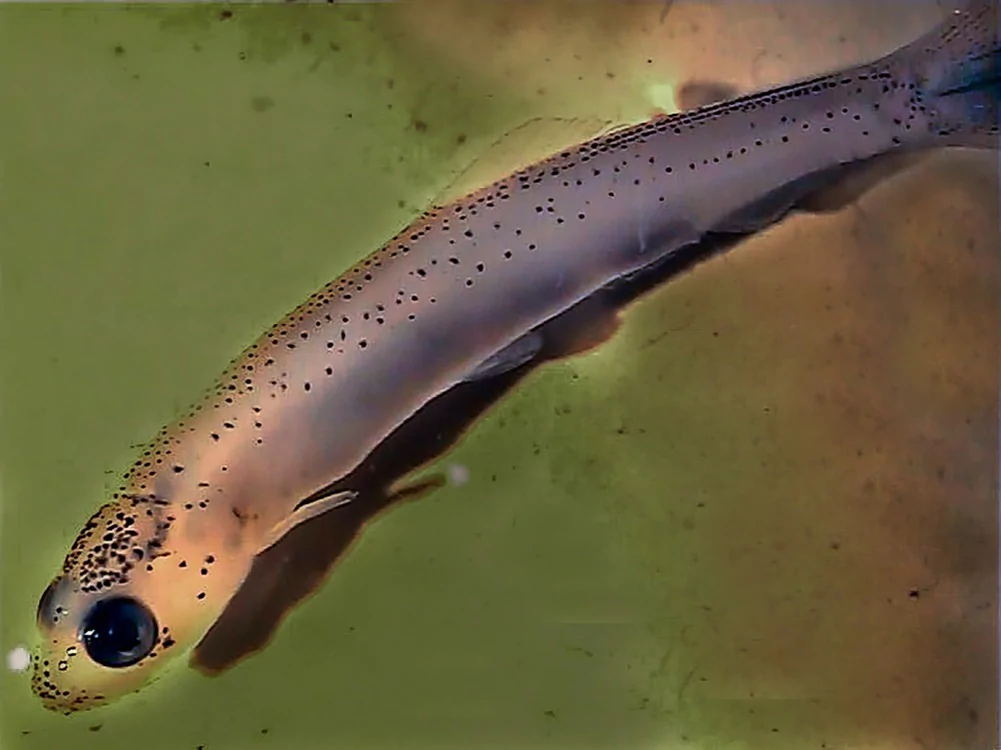
*A. fallax* larva observed through stereomicroscope. The specimen corresponds to the post‐flexion stage. Measured parameters include larval length, length‐to‐height ratio, chromatophore number and position and eye diameter.

Stereomicroscope analysis (Figure 4) revealed that the shad larvae had a length ranging from 17 to 22 mm, with an average length of 19.5 mm and a length‐to‐height ratio of approximately 8.3. The number of chromatophores was counted, showing a variable range from 800 to 1200 depending on the size of the specimen analysed. Three main chromatophore positions were identified: periopercular, dorsal and frontal. The eye diameter was measured and found to vary between 0.85 and 1 mm.

The *Cytb* gene fragments, analysed in 15 shad larvae, were aligned unambiguously for 420 bp cutting the 50–30 extremes to delete bases with low sequencing resolution (about 95 bp). The samples presented the same single‐nucleotide polymorphism (SNPs) profile of the previously described *A. fallax* hap3 (Genbank A.N. JF681128, see Chiesa et al., 2014) found in the Taro River and Po River. In relation to this, *A. fallax* hap3 was already described in other landlocked and anadromous Italian populations, confirming the genetic continuity of this haplotype across the Taro and Po River systems. (Chiesa et al., 2014).

## 4 DISCUSSION

The twaite shad (*A. fallax*), an anadromous species of the Clupeidae family, is facing significant population declines across Europe, mainly due to anthropogenic pressures such as migration barriers, pollution, habitat degradation, predation by invasive species and unsustainable fishing (Freyhof et al., 2008; King & Roche, 2008; Mattocks et al., 2017). These factors collectively disrupt the lifecycle and ecological balance of *A. fallax*, posing serious conservation concerns (Aprahamian et al., 2003). The migration and spawning behaviour of *A. fallax* is a critical aspect of its life cycle, as the demersal eggs hatch relatively quickly and the larvae begin a planktonic life in the river. This period is crucial for the survival and development of the species, and any disruptions during this phase can have profound impacts on population sustainability (Aprahamian et al., 2003).

Our study applied environmental DNA (eDNA) techniques to detect the presence of *A. fallax* in various sites within the Po River and Taro River. The use of eDNA, coupled with Illumina sequencing of the 12S rRNA gene, allowed for a sensitive and accurate assessment of the species' distribution during the spawning period. The detection of 26 different species across the sites confirmed high biodiversity ranges within these aquatic ecosystems and highlighted the efficacy of eDNA analysis in capturing a broad range of species. In particular, eDNA analysis revealed the presence of *A. fallax* at both Sites ST and CT, located within the Taro River. These findings indicated that these sites serve as active spawning grounds for the species. The detection of *A. fallax* eDNA at these locations supported the hypothesis that the Taro River provides suitable spawning habitats. However, it was notably absent during the second sampling at Site ST (ST_2). This variability highlights the temporal dynamics of *A. fallax* presence and underscores the importance of repeated sampling for accurate monitoring. The absence of *A. fallax* eDNA in ST_2 likely reflects the influence of hydrological and meteorological variability, particularly turbidity and water volume fluctuations associated with spring floods and heavy rainfall. These conditions not only affect species behaviour but also limit the efficiency of eDNA detection, as excessive turbidity can rapidly clog filters and promote DNA adsorption onto suspended particles, reducing recoverable DNA in the water column. Conversely, periods of lowering water volume following flood peaks can lead to increased sedimentation and localized DNA degradation. Together, these dynamics contribute to a reduced probability of *A. fallax* detection.

Our model identified conductivity as the environmental variable most strongly associated with *A. fallax* occurrence, with a marked increase in detection probability around ~350 μS/cm. This pattern suggests a relationship between conductivity, turbidity and hydrological regime, as all tend to fluctuate with flood and post‐flood conditions typical of the species' spring spawning period. Conductivity in riverine systems reflects both natural ionic inputs (e.g., weathering, groundwater) and anthropogenic sources (e.g., agricultural runoff). Moderate conductivity values may indicate well‐oxygenated, nutrient‐balanced conditions favourable for spawning and early development, whereas extreme values could signal stress from pollution or oligotrophy. We hypothesize that *A. fallax* may select habitats within an optimal conductivity range corresponding to suitable hydrological and physicochemical conditions during its reproductive migration. This association highlights the importance of integrating abiotic predictor (such as conductivity, turbidity and water level dynamics) into future monitoring and habitat suitability models for diadromous species.

Conversely to Taro sites, eDNA analysis did not detect *A. fallax* in two surveyed sites within the Po River, designated as DP (Downstream Po) and UP (Upstream Po). The absence of shad DNA at these sites suggested that these locations may not currently support spawning activities for *A. fallax*. One critical factor could account for this absence: the timing of our sampling. The sampling was conducted at the end of April and the beginning of May, which might have been too early to detect *A. fallax* in these sites. According to previous publications (Chiesa et al., 2014; Sabatino et al., 2022), the presence of twaite shad in the Po River main course is typically noted in July as a probable return to the sea. This suggests that the species may not be present at these sites by the time of our sampling that would represent only a migration corridor without specific stationing in spawning sites. Additional investigations are required to test this hypothesis. To validate the specificity and accuracy of our eDNA detection method, we included an outgroup site (CE) where *A. fallax* is known to be absent. As expected, eDNA analysis did not detect *A. fallax* in the outgroup site where the species is absent, supporting the reliability of our eDNA methodology. The species' absence in Site CE reinforced the confidence in our positive detections at Sites ST and CT and the negative results at Sites DP and UP. The presence of eDNA at ST site was further validated by morphological identification of larvae, confirming that these habitats are essential for the reproductive success of *A. fallax*.

In combination with eDNA investigations, we conducted scuba diving observations to directly monitor the spawning activity of *A. fallax* at suitable sites previously identified through eDNA analysis. The aim of scuba diving visual census was to identify the reproductive sites on a microgeographic scale. These observations were performed only at Site ST, despite both Site ST and Site CT showing the presence of *A. fallax* via eDNA. Although eDNA analysis confirmed the presence of *A. fallax* at Site CT, it was excluded from scuba diving approaches due to its not appropriate substrate composition. In fact, Site CT consists of sand and silt, which are not conducive to the reproductive deposition phase of *A. fallax*. Fine sediments can lead to reduced oxygen levels and increased turbidity, which are detrimental to egg viability (Corell et al., 2023). Such substrate is not suitable for the reproductive deposition phase of *A. fallax*, which typically requires gravel substrates with better oxygenation and stability for successful egg attachment and development in the interstitial frames. Contrarily, Site ST possesses a gravelly substrate, which is ideal for the spawning of *A. fallax*. In fact, this kind of substrate provides several critical benefits for the reproductive phase: oxygenation, stability and protection. The interstitial spaces allow for better water flow and oxygenation, which are essential for egg development: The rough texture of gravel provides a protective environment for eggs against predation and physical disturbances. (Corell et al., 2023; Frankham, 2005).

The stereomicroscopic analysis of shad larvae collected at the ST site, corresponding to the post‐flexion developmental stage, revealed a length range of 17–22 mm, with an average length of 19.5 mm, which is consistent with the averages reported in previous studies (Esteves & Pedro Andrade, 2008; Gerkens & Thiel, 2001). These morphometric parameters are crucial for understanding the early development stages of *A. fallax* and can serve as baseline data for future studies. The chromatophore count, varying between 800 and 1200, depending on the specimen size, provided additional information on the pigmentation and potential camouflage strategies of the larvae. Such pigmentation could be vital for survival, offering protection from predators during the vulnerable early life stages. The eye diameter of the larvae, ranging from 0.85 to 1 mm, was another important metric, indicating visual development which is crucial for feeding and predator avoidance. The identification of three main chromatophore positions, periopercular, dorsal and frontal, added to our understanding of the species' morphological adaptations. These positions likely play a role in species‐specific signalling and camouflage, essential for both intra‐species communication and predator avoidance (R., 2000). The same larvae observed through microscopy were subjected to *Cytb* genetic analysis for accurate species identification. The results of the DNA barcoding analysis confirmed that all the larvae were indeed *Alosa fallax*, providing conclusive evidence of the species being present. Moreover, these larvae were found to belong to the mitochondrial haplotype hap3 previously described in the Taro River by Chiesa et al. (Chiesa et al., 2014). Given this background, the use of *Cytb* in our study ensures methodological continuity and comparability with prior genetic assessments of *A. fallax* in the study area. While we are aware that sequencing the 12S region from larvae could also have represented a valuable complementary approach, particularly for direct comparison with eDNA metabarcoding datasets, the current strategy has relied on a marker with demonstrated discriminatory power and historical relevance for this species in Italian inland and transitional waters. This finding not only validates the morphological observations but also aligns with the genetic profiles documented in earlier studies, reinforcing the reliability of our current assessment and the historical data on *A. fallax* populations in the Taro River.

The integration of eDNA analysis with scuba diving observations and molecular barcoding has proven to be a powerful and effective approach for identifying spawning sites of *Alosa fallax* in the Taro River. This multi‐level framework combines non‐invasive genetic detection with direct field verification and precise species identification, enabling comprehensive and reliable monitoring. eDNA analysis offers a sensitive and efficient tool for detecting *A. fallax* presence, even when individuals are not directly observable, and is particularly useful for large‐scale preliminary screening of potential spawning habitats. Scuba diving surveys provide in situ confirmation of eDNA results by documenting spawning behaviour and habitat features, ensuring that molecular detections reflect actual biological activity. Cytb barcoding further validates species identity and contributes to understanding population structure and genetic diversity. This genetic validation is crucial for ensuring the accuracy of the species identification and understanding the genetic diversity within populations. Together, these methods form a robust framework for assessing and monitoring *A. fallax* spawning sites. This sampling approach is not only effective for the Taro River but can also be applied to other watercourses that are potential reproductive zones where *A. fallax* has not yet been described. By using this integrated approach, conservationists and researchers can better identify critical habitats, track population dynamics and implement targeted conservation strategies to protect and enhance *A. fallax* populations across Europe.

Although effective, our sampling design presents some limitations. The restricted temporal coverage may not fully capture the spawning dynamics of *A. fallax*, and environmental factors such as turbidity, flow and temperature could influence eDNA persistence and detection, potentially leading to false negatives. Expanding temporal replication would enhance the robustness of future assessments.

In conclusion, our study has provided significant insights into the spawning behaviour and larval development of the twaite shad, *Alosa fallax*, in the Po River basin. By employing a multi‐level framework that integrates environmental DNA (eDNA) techniques with traditional observational methods, we have effectively captured a comprehensive snapshot of this anadromous species' migration and spawning activities in Taro sites.

## AUTHOR CONTRIBUTIONS

Conceptualisation: AA, LF, FNM. Developing methods: AA, LF, MAC, MS, MEA. Data analysis: NB, AA, MAC, PMR. Preparation of figures and tables: AA, NB, PMR. Conducting the research, data interpretation, writing: AA, LF, NB, MAC, MS, AME, DN, AP, RP, FNM.

## FUNDING INFORMATION

This research was carried out within the framework of the project Ministry of University and Research (MUR), Italy “Fishing 4 Biodiversity” (NBFC_S8P1_0067)—“Biodiversity of the Po River Basin Water Bodies”, Work Package 1 “Monitoring of Target Species”. The activities were funded through the service agreement assigned to Gen Tech S.r.l.

## CONFLICT OF INTEREST STATEMENT

The authors declare that they have no known competing financial interests or personal relationships that could have appeared to influence the work reported in this paper.

## Supporting information


**Figure S1.** Intensity per base and quality score distribution across sequencing cycles. Data by cycle (left panel) display the intensity values for each nucleotide (A, C, G, T) over sequencing cycles, with both surfaces and all channels combined. Peaks indicate signal quality, with average intensities of A = 309.79, C = 1097.89, G = 276.32 and T = 390.61. QScore distribution (right panel) shows the quality score distribution of the reads, highlighting that 7.17G reads (0.68%) exceed Q30, indicating high sequencing accuracy across cycles.
**Figure S2**. Cluster density and quality score heat map across sequencing lanes. Data by lane (left panel) illustrate the density of clusters in k/mm^2^ across sequencing lanes, including both surfaces. The blue lines represent overall density, while the green lines indicate pass‐filter (PF) clusters. Average density values are consistent across lanes. QScore heat map (right panel) shows the distribution of quality scores (QScores) over sequencing cycles for both surfaces. The heat map uses a colour gradient, where red indicates lower‐quality scores and green indicates higher‐quality scores. Most cycles maintain high‐quality scores (*Q* > 30), ensuring reliable sequencing data.

## Data Availability

The data used in this article are available upon request from the corresponding author.
