# Supplementary material for: Integrating environmental DNA, diving surveys and DNA barcoding to track endangered twaite shad ( A losa fallax) spawning sites
Source: J Fish Biol. 2026 May 25;109(2):1198–209. doi: 10.1111/jfb.70489 (PMC13521689; doi:10.1111/jfb.70489)
Supplement: Supplementary file 1 [file JFB-109-1198-s001.docx]

# **Supporting information**

**S1 Fig. Intensity Per Base and Quality Score Distribution Across Sequencing Cycles**. Data by cycle (left panel) displays the intensity values for each nucleotide (A, C, G, T) over sequencing cycles, with both surfaces and all channels combined. Peaks indicate signal quality, with average intensities of A = 309.79, C = 1097.89, G = 276.32, and T = 390.61.
QScore distribution (right panel) shows the quality score distribution of the reads, highlighting that 7.17G reads (0.68%) exceed Q30, indicating high sequencing accuracy across cycles.

**S2 Fig. Cluster Density and Quality Score Heatmap Across Sequencing Lanes.** Data by lane (left panel) illustrates the density of clusters in k/mm² across sequencing lanes, including both surfaces. The blue lines represent overall density, while the green lines indicate pass-filter (PF) clusters. Average density values are consistent across lanes. QScore heatmap (right panel) shows the distribution of quality scores (QScores) over sequencing cycles for both surfaces. The heatmap uses a color gradient, where red indicates lower-quality scores and green indicates higher-quality scores. Most cycles maintain high-quality scores (Q > 30), ensuring reliable sequencing data.
